# OXA-48 Carbapenemase-Producing Salmonella typhimurium Nosocomial Bacteremia in the Intensive Care Unit: A Case Report and Review of the Literature

**DOI:** 10.7759/cureus.35811

**Published:** 2023-03-06

**Authors:** Fayrouz Debbagh, Fayçal Idam, Asmae Lamrani Hanchi, Nabila Soraa

**Affiliations:** 1 Department of Microbiology, Arrazi Hospital, Mohammed VI University Hospital, Marrakesh, MAR

**Keywords:** blaoxa-48, carbapenemase, intensive care unit, surgical wound infection, nosocomial infection, bacteremia, salmonella typhimurium

## Abstract

*Salmonella enterica serovar Typhimurium* is a gram-negative bacterium mainly involved in foodborne diseases. Several pathways of antimicrobial resistance have been recently identified in this strain. This article reports a case of a patient hospitalized in intensive care who underwent emergency trauma surgery. During his hospitalization, he developed a nosocomial bacteremia from a surgical wound infection. The cytobacteriological examination of the surgical site and the blood culture isolated *Salmonella spp.* susceptible to third-generation cephalosporins, resistant to ertapenem, and with decreased sensitivity to imipenem. The carbapenemase test was positive for blaOXA-48. The serotyping identified *Salmonella enterica serovar Typhimurium*. The patient's response to antibiotics was favorable.

## Introduction

Enzymatic hydrolysis of beta-lactam is the most common mode of resistance for this class of antibacterial agents in clinically important gram-negative bacteria. Carbapenemases are enzymes that are partly responsible for the resistance described in the family of *Enterobacteriaceae*, and are divided into three classes (class A, class B, and class D oxacillinases), the most frequent of which is OXA-48. This blaOXA-48 gene is generally detected in *Klebsiella pneumoniae* and *Escherichia coli,* but can also be found in the other *Enterobacteriaceae* [1].

In recent years, several studies on the *Salmonella *genus have been published, focusing on the increasing number of isolates carrying different carbapenemase genes. The emergence of multidrug-resistant *Salmonella *strains is of particular concern for the *Salmonella enterica* species, which causes infections of increasing severity requiring rational and specific management [2]. The variety of resistance mechanisms, as well as the existence of a considerable number of multidrug-resistant *Salmonella enterica* strains, represent a significant threat to public health and may be responsible for the silent spread of potentially difficult-to-treat strains, demanding attentive control.

This article reports a case of an intensive care unit patient with OXA-48 carbapenemase-producing *Salmonella typhimurium* bacteremia that was susceptible to third-generation cephalosporins.

## Case presentation

In February 2021, a 20-year-old man with no prior medical or surgical history was hospitalized in the intensive care unit of the Mohammed VI University Hospital of Marrakesh for polytrauma caused by a road accident. The impact points were the lower limbs and the craniofacial region. He had emergency surgery for a fracture of the right femur and an open fracture of the left leg. He was sedated, intubated, and ventilated. After surgery, the patient was given prophylactic antibiotics consisting of 4 grams of piperacillin-tazobactam every eight hours.

Throughout his hospitalization, the patient developed multiple infections with mostly multidrug-resistant bacteria, including nosocomial pneumonia with *Escherichia coli* and *Staphylococcus aureus* methicillin-resistant strain, bacteremia with *Enterococcus faecium*, and an infection of the amputation stump of the left leg with three multidrug-resistant bacteria, including *Escherichia coli*, *Klebsiella pneumoniae,* and *Enterobacter cloacae*. The patient received linezolid 0.6 grams twice daily.

**Figure 1:**
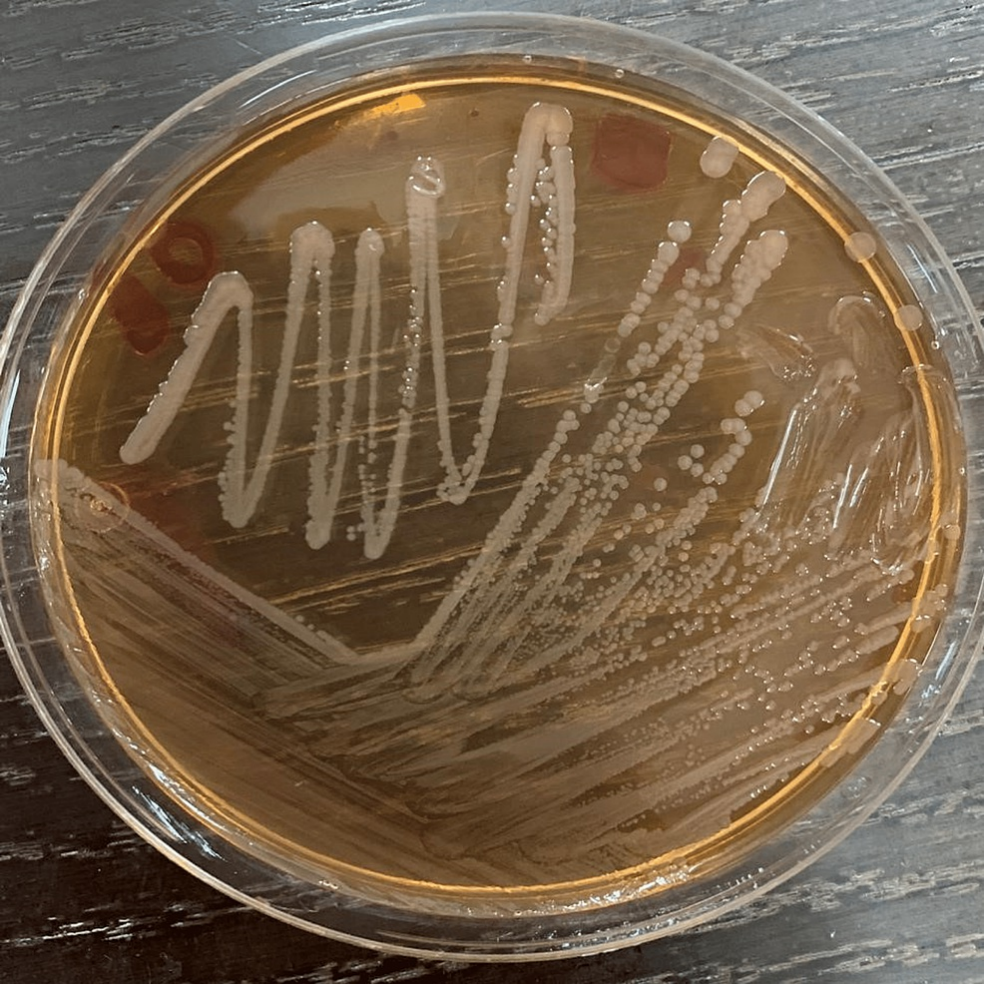
Non-lactose-fermenting colonies of Salmonella typhimurium on the MacConkey medium

**Figure 2:**
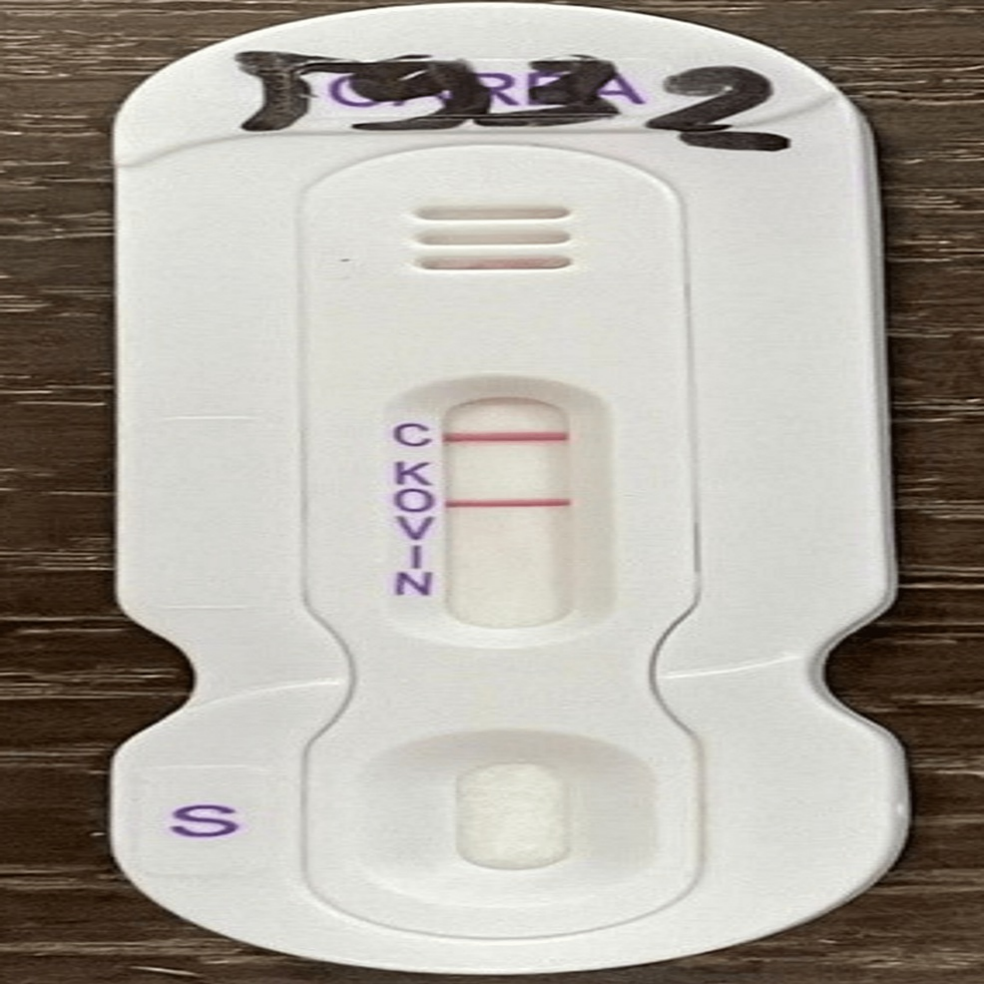
BlaOXA-48-positive carbapenemase testing of Salmonella typhimurium using the NG-Test Carba 5

On the 26th day of his hospitalization, the patient had an infectious syndrome with a C-reactive protein (CRP) of 75 mg/L and hyperleukocytosis of 22,440/mm^3^, mostly neutrophilic; etiological investigations were initiated. Blood culture and cytobacteriological examination of pus from the amputation stump yielded the identification of *Salmonella spp.* by matrix-assisted laser desorption ionization-time of flight (MALDI-TOF) mass spectrometry (Figure 1). Minimum inhibitory concentration (MIC) determination on the liquid medium was performed on a BD Phoenix M50 (BD Diagnostics, Sparks, MD). The *Salmonella spp.* strain was susceptible to third-generation cephalosporins with a decreased sensitivity to carbapenems represented by resistance to ertapenem and an intermediate sensitivity to imipenem. The presence of blaOXA-48 was determined using the NG-Test Carba 5 (NG Biotech, Guipry, France) chromatographic testing for carbapenemase (Figure 2). Serotyping using the White-Kauffmann-Le Minor (WKL) scheme based on serological detection identified *Salmonella enterica subsp. enterica*, serotype *Typhimurium*.

Table 1 summarizes the antibiotic sensitivity of the *Salmonella typhimurium* strain.

**Table 1:** Antibiotic susceptibility of Salmonella typhimurium by MIC calculation on liquid medium According to the European Committee on Antimicrobial Susceptibility Testing (EUCAST) criteria [3]. MIC: minimum inhibitory concentration; R: resistant; I: intermediate; S: susceptible.

Antibiotic tested	MIC (µg/ml)
Amoxicillin	>16 (R)
Amoxicillin-clavulanate	>32/2 (R)
Cefazolin	>32 (R)
Cefixime	≤0.5 (S)
Ceftriaxone	≤1 (S)
Ceftazidime	≤1 (S)
Piperacillin-tazobactam	>16 (R)
Ertapenem	>2 (R)
Imipenem	4 (I)
Ciprofloxacin	≤0.25 (S)
Levofloxacin	≤0.5 (S)
Trimethoprim-sulfamethoxazole	≤2 (S)
Gentamicin	>4 (R)
Amikacin	>16 (R)
Tobramycin	>4 (R)
Tigecycline	≤1 (S)

Empiric anti-biotherapy was initiated for nosocomial bacteremia, including imipenem 3 g/day, amikacin 1.5 g/day, and vancomycin 2 g/day, with a positive evolution.

## Discussion

In contrast to other *Enterobacteriaceae* (*Klebsiella pneumoniae and Escherichia coli*), non-typhi *Salmonella *has very rarely been described as resistant to carbapenems. The presence of beta-lactamase with or without selective impermeability by modification of porins remains the main mechanism of this resistance. Five carbapenemases have been reported in non-typhi *Salmonella*: KPC (*Klebsiella pneumoniae* carbapenemases, class A), IMP (imipenemase), NDM (New Delhi metallo-β-lactamase), VIM (Verona integron-encoded metallo-β-lactamase, class B), and OXA-48 (oxacillinase, class D) [4].

The first case of OXA-48 carbapenemase-producing *Salmonella* was described in France in 2009 by Hello et al. [2] in a 69-year-old female patient returning from Egypt, found on blood culture and stool samples. The strain was identified as *Salmonella Saintpaul* with intermediate sensitivity to imipenem and carried the *blaOXA-48* gene on an IncL/M conjugative plasmid of an estimated size of 70 kb. *Salmonella Kentucky* ST198-X1 was isolated from the feces of the same patient in 2011; it was resistant to third-generation cephalosporins, ciprofloxacin, and azithromycin, and was producing a blaOXA-48 carbapenemase with in vitro susceptibility to imipenem. In 2012, a perianal swab screening of a patient transferred from Libya to Switzerland identified a second case of multidrug-resistant OXA-48-producing *Salmonella Kentucky *ST198 with decreased imipenem sensitivity [5]. The third case was reported in 2013, where two isolates of *S. Paratyphi B* and *S. typhimurium* carrying OXA-48 carbapenemase genes were detected in two patients hospitalized for diarrhea in the United Kingdom, one of whom had been to Africa. Antibiotic sensitivity testing revealed intermediate ertapenem and cefotaxime sensitivity, with preserved susceptibility to imipenem and ceftazidime. The blaOXA-48 gene was located on a 62 kb IncL/M plasmid [6]. In 2020, *Salmonella Kentucky* was isolated in Saudi Arabia from the stool of a Sudanese patient. This strain was a multidrug-resistant carbapenemase OXA-48 producer, with decreased sensitivity to tigecycline while being susceptible to all aminoglycosides and colistin [7]. In Morocco, a study published in 2013 by Hello et al. identified 226 *Salmonella *strains, of which 30 (13%) were of the *Salmonella Kentucky* serotype. Only five isolates showed decreased sensitivity to carbapenems with the detection of the blaVIM-2 gene. No production of carbapenemase type OXA-48 was described [2].

This article reports the first case of nosocomial bacteremia caused by an OXA-48 carbapenemase-producing *Salmonella typhimurium* from a surgical site origin. *Salmonella enterica Typhimurium* is mostly involved in food poisoning and septicemia in young children. A growing proportion of nosocomial infections and poisonings are attributable to the bacterium's high virulence and antibiotic resistance [8]. However, this strain is rarely implicated in infections of surgical sites [9]. In 2018, two strains of *Salmonella typhimurium* were isolated in two children in China, one of whom developed a surgical wound infection after emergency trauma surgery [9]. They were both susceptible to carbapenems, quinolones, and macrolides but resistant to third- and fourth-generation cephalosporins. Several studies suggest that the recent increase in cephalosporin-resistant *Salmonella typhimurium* is related to various antibiotic treatments [10]. Multidrug resistance of *Salmonella *strains was particularly noted in *S. typhimurium* isolates, whose spread is supported by the dissemination of dominant resistant clones [10]. Even though this strain is sensitive to third-generation cephalosporins and other antibiotics, the probabilistic antibiotic therapy that was started with imipenem for the reported case was continued due to the good local and general evolution.

It is important to mention that the spread of *Salmonella typhimurium* is essentially through fecal-oral transmission. However, its recent presence in the hospital environment and its isolation in surgical wounds suggest that the contamination of the hospital environment may constitute a reservoir of the bacterium and be responsible for nosocomial infections, as it is reported [9].

In terms of carbapenem resistance, OXA enzymes (oxacillin hydrolyzing carbapenemases) represent class D carbapenemases. OXA-48 is the most isolated carbapenemase in its class and is often detected within *Klebsiella pneumoniae* [11]. The enzymatic kinetics of OXA-48 revealed that its hydrolytic activity is high against penicillins but low against carbapenems [12]. As a result, OXA-48 hydrolyzes imipenem and meropenem less effectively than ertapenem, which is this enzyme's optimal substrate [13].

In several countries, the number of research studies on the generation of OXA-48 by carbapenemase-producing *Enterobacteriaceae* (CPE) has increased in recent years [13]. In fact, these OXA-48-generating isolates have become more prevalent than those producing the traditional carbapenemases KPC, NDM, and VIM [4]. This dissemination could be explained by the genetic support of blaOXA-48, which is generally located on transposons of the Tn1999 type, carried by self-transferable plasmids of about 60 kb belonging to the IncL/M incompatibility group. They are mainly responsible for its widespread dissemination among *Enterobacteriaceae *[12]. Thus, the spread of blaOXA-48-producing strains is most likely linked to a high level of human population exchange with endemic areas, including Turkey, the Middle East, and North African countries (mainly Morocco, Tunisia, Egypt, and Libya) [14,15]. In *Salmonella *species, horizontal transfer of carbapenemase-encoding genes as well as co-adaptations and selective cross-adaptations would probably be involved in the development of carbapenem resistance by non-typhi​​​​​​​ *Salmonella* [4].

## Conclusions

The increasing number of studies regarding CPE demonstrate how resistant *Enterobacteriaceae *are evolving, resulting in a significant threat to the bacterial environment. Although cases of nosocomial infection due to OXA-48-carbapenemase-producing *Salmonella typhimurium* are uncommon, they highlight the need for heightened vigilance in hospitals. Therefore, it is necessary to increase monitoring and intra-hospital safety mechanisms to prevent the spread of this potentially lethal, multi-resistant bacteria and to highlight the need for hygiene and sterilization prior to any medical or surgical treatment.

## References

[1] Bush K, Jacoby GA (2010). Updated functional classification of β-lactamases. Antimicrob Agents Chemother.

[2] Le Hello S, Harrois D, Bouchrif B (2013). Highly drug-resistant Salmonella enterica serotype Kentucky ST198-X1: a microbiological study. Lancet Infect Dis.

[3] EUCAST. 2013 (2023). European Committee on Antimicrobial Susceptibility Testing. Breakpoint tables for interpretation of MICs and zone diameters. https://www.eucast.org/fileadmin/src/media/PDFs/EUCAST_files/Breakpoint_tables/v_13.0_Breakpoint_Tables.pdf.

[4] Fernández J, Guerra B, Rodicio MR (2018). Resistance to carbapenems in non-typhoidal salmonella enterica serovars from humans, animals and food. Vet Sci.

[5] Seiffert SN, Perreten V, Johannes S, Droz S, Bodmer T, Endimiani A (2014). OXA-48 carbapenemase-producing Salmonella enterica serovar Kentucky isolate of sequence type 198 in a patient transferred from Libya to Switzerland. Antimicrob Agents Chemother.

[6] Day MR, Meunier D, Doumith M, de Pinna E, Woodford N, Hopkins KL (2015). Carbapenemase-producing Salmonella enterica isolates in the UK. J Antimicrob Chemother.

[7] Alghoribi MF, Desin TS, Alswaji AA (2020). OXA-48 carbapenemase-producing Salmonella enterica serovar Kentucky ST198 isolated from Saudi Arabia. J Antimicrob Chemother.

[8] Yang C, Li H, Zhang T, Chu Y, Zuo J, Chen D (2020). Study on antibiotic susceptibility of Salmonella typhimurium L forms to the third and forth generation cephalosporins. Sci Rep.

[9] Qin H, Guo Y, Li Y, Zheng R (2020). Molecular relatedness of Salmonella enterica Typhimurium isolates from feces and an infected surgical wound. Infect Drug Resist.

[10] Fàbrega A, Vila J (2013). Salmonella enterica serovar Typhimurium skills to succeed in the host: virulence and regulation. Clin Microbiol Rev.

[11] Smith HZ, Kendall B (2022). Carbapenem Resistant Enterobacteriaceae. https://www.ncbi.nlm.nih.gov/books/NBK551704/.

[12] Poirel L, Potron A, Nordmann P (2012). OXA-48-like carbapenemases: the phantom menace. J Antimicrob Chemother.

[13] Mairi A, Pantel A, Sotto A, Lavigne JP, Touati A (2018). OXA-48-like carbapenemases producing Enterobacteriaceae in different niches. Eur J Clin Microbiol Infect Dis.

[14] Martínez-Martínez L, González-López JJ (2014). Carbapenemases in Enterobacteriaceae: types and molecular epidemiology. Enferm Infecc Microbiol Clin.

[15] Stewart A, Harris P, Henderson A, Paterson D (2018). Treatment of infections by OXA-48-producing Enterobacteriaceae. Antimicrob Agents Chemother.

